# Occult cement plug causing femoral canal obstruction during nailing after TKA: a case report

**DOI:** 10.3389/fsurg.2026.1939276

**Published:** 2026-08-24

**Authors:** Yanhui Zhang, Hongliang Song, Bingxiang Yan, Mingkuan Shao, Fang Yuan, Xin Nie

**Affiliations:** 1 Department of Orthopedic Surgery, The First Affiliated Hospital of Shandong First Medical University & Shandong Provincial Qianfoshan Hospital, Jinan, China; 2 Department of Orthopedics, Feicheng Hospital of Traditional Chinese Medicine, Taian, Shandong, China; 3 Department of Orthopedics, Huantai County People’s Hospital, Zibo, Shandong, China; 4 Department of Oncology and Hematology, Dongping County People’s Hospital, Taian, Shandong, China

**Keywords:** bone cement plug, case report, femoral shaft fracture, intramedullary nailing, total knee arthroplasty

## Abstract

With the aging global population, periprosthetic and post-arthroplasty femoral shaft fractures are becoming increasingly common. Although intramedullary nailing remains one of the classic surgical procedures for treating femoral shaft fractures in many cases, the presence of an unexpected space-occupying lesion within the medullary canal may often increase the surgical difficulty. This paper reports a case of a femoral fracture occurring 10 years after ipsilateral total knee arthroplasty in an 83-year-old female. On preoperative plain radiographs and CT, no clear intramedullary lesion was identified. However, during the subsequent fracture surgery, a dense polymethyl methacrylate (bone cement) plug was encountered that completely blocked reaming and nail insertion. The surgery was ultimately completed by using a side plate for fixation. This case indicates that radiolucent cement plugs retained from prior joint surgery may be missed on preoperative imaging. Orthopedic surgeons must therefore maintain a high index of suspicion and always have alternative fixation strategies available to manage such intraoperative crises effectively.

## Introduction

With the continuous aging of the global population and the corresponding rise in the volume of primary total knee arthroplasties (TKA), related orthopedic complications have increased significantly (1). Among these, fractures adjacent to or above a knee replacement site represent a severe clinical challenge for joint and trauma surgeons alike (2). As the cohort of patients living with long-standing arthroplasties expands annually, the incidence of these complex periprosthetic or post-arthroplasty fractures has steadily risen, demanding robust surgical strategies and meticulous preoperative planning (3).

Femoral shaft fractures constitute approximately 6% of all skeletal fractures, with the vast majority occurring in the middle and lower segments of the femur (4). Currently, intramedullary nailing is still a common treatment for femoral shaft fractures, due to its biomechanical benefits as a load-sharing device and its preservation of the biological environment of the fracture hematoma (5). However, the success of this technique relies entirely on the patency and structural integrity of the femoral medullary canal (6).

We report a rare case of an 83-year-old woman with a femoral fracture and a history of ipsilateral total knee arthroplasty performed a decade earlier, in which a previously unrecognized cement plug was discovered in the medullary canal during surgery. It is noteworthy that no definite intramedullary lesion was identified on preoperative imaging studies, including high-resolution plain radiography and computed tomography (CT). This report aims to analyze the diagnosis, clinical reasoning, and intraoperative crisis management associated with this case, while critically evaluating the hidden “pitfalls” of standard preoperative imaging in patients with a history of joint arthroplasty.

## Case report

An 83-year-old female was admitted to our emergency department presenting with severe pain, visible deformity, and total mobility restriction in her left thigh lasting for one day. The symptoms were triggered by an accidental slip and fall at home, during which she landed directly on her left knee. She immediately experienced acute pain and was unable to stand or bear weight. Her past surgical history was notable for a primary left TKA performed ten years prior at an external institution in the United States due to severe, end-stage osteoarthritis of the left knee. Following that procedure, her functional recovery had been highly acceptable, and she had successfully resumed independent ambulation without assistive devices. The patient denied any other major medical comorbidities or additional surgical history, and she had no history of long-term corticosteroid or bisphosphonate therapy.

Upon admission, a detailed physical examination was performed. Local findings revealed significant swelling, ecchymosis, and an obvious deformity in the middle and lower sections of the left thigh, accompanied by palpable bone crepitus and abnormal segment movement. On neurovascular evaluation, the distal circulation was completely intact; the pulsation of the left dorsalis pedis artery was strong and palpable, and there were no sensory or motor deficits in the left toes or foot.

**Figure 1:**
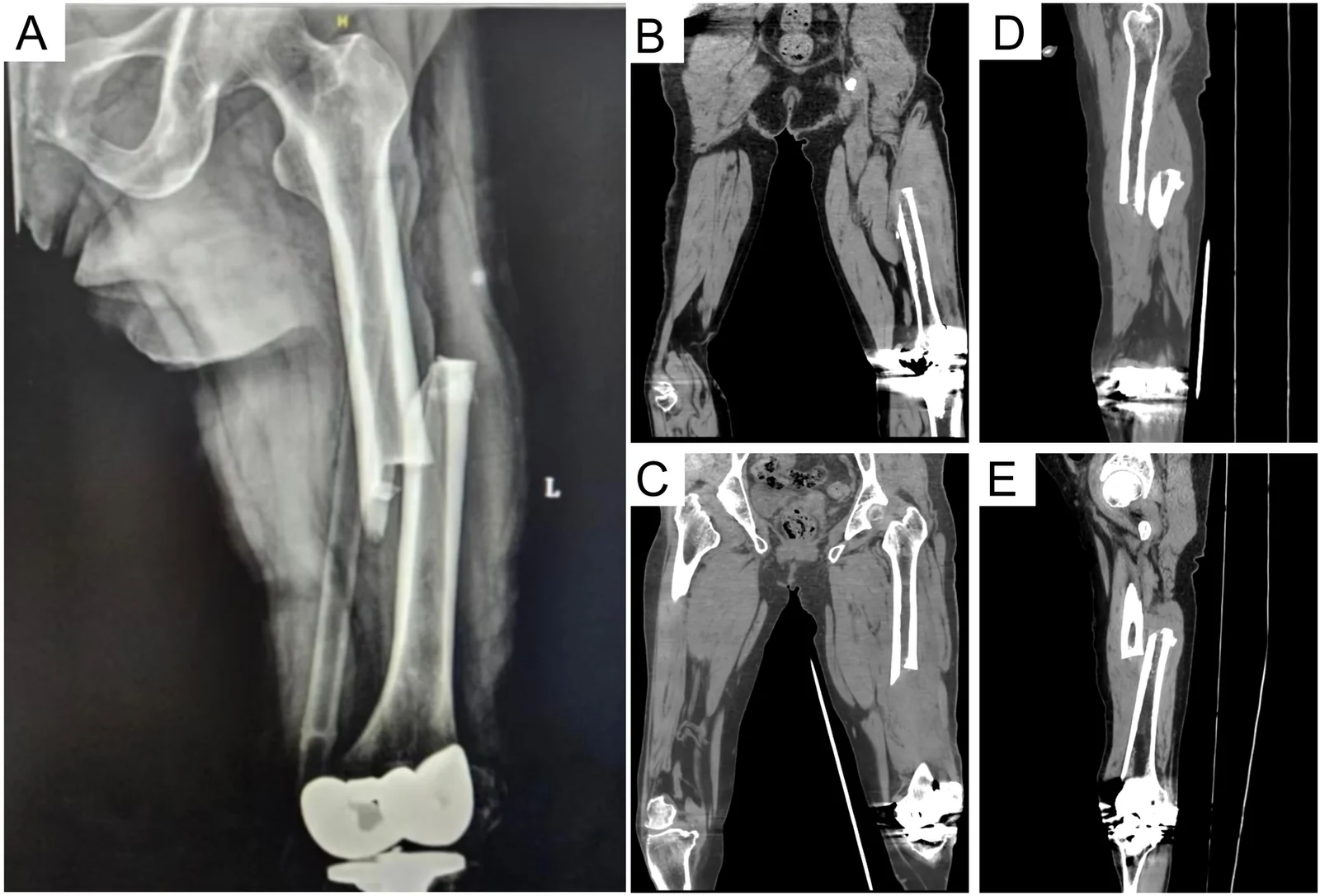
Preoperative imaging of the left femur. **(A)** Anteroposterior full-length radiograph of the left femur. **(B,C)** Coronal high-resolution CT images. **(D,E)** Sagittal high-resolution CT images.

Emergency imaging assessment comprised full-length AP and lateral plain radiographs of the left femur, along with coronal and sagittal high-resolution computed tomography (CT) scans (Figures 1A-E). Imaging showed a mid-diaphyseal femoral fracture with a classic transverse morphology, associated with focal lateral cortical thickening and a small medial spur. According to the ASBMR criteria and after exclusion of high-energy trauma, the fracture was definitively diagnosed as an atypical femoral fracture (AFF) type II (complete fracture). On x-ray and CT, no definite radiopaque foreign bodies, bone cement columns, or space-occupying lesions were identified in the distal or proximal medullary canal. Given the patient's advanced age, independent baseline functional status, and the specific transverse fracture geometry, a multi-disciplinary departmental discussion concluded that intramedullary nailing was the optimal intervention.

**Figure 2:**
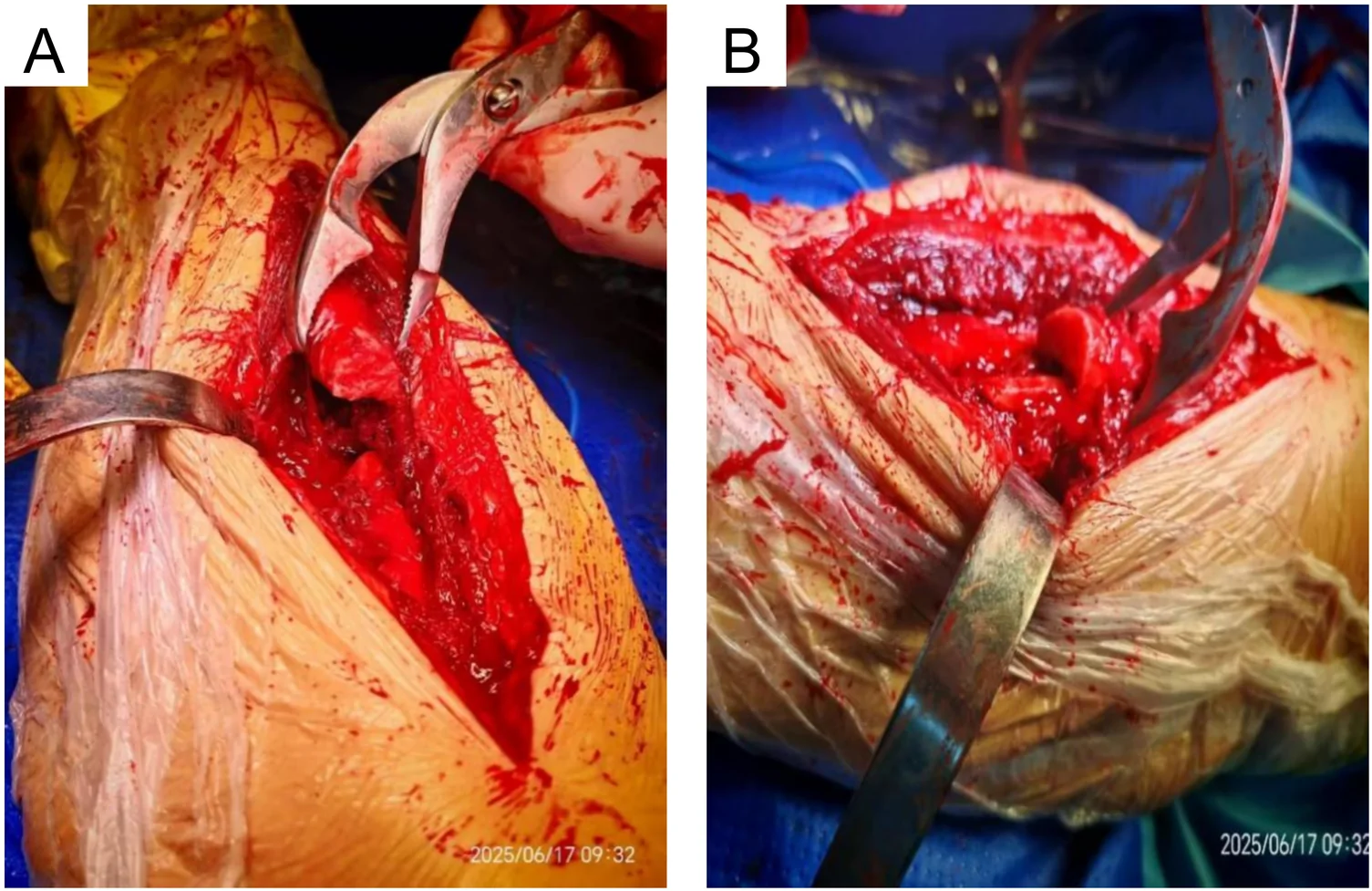
Intraoperative visual findings through the fracture site. **(A,B)** During preparation for progressive intramedullary reaming, a solid, spindle-shaped, high-density mass (bone cement plug) was encountered, completely occluding both the proximal and distal dimensions of the medullary canal.

On day 3 of admission, the patient was taken to the operating theater, and the procedure was performed under combined spinal-epidural anesthesia. However, an unexpected situation was encountered during the attempted intramedullary nailing: the reamer met a solid, unyielding obstruction within the distal medullary lumen. Upon direct visual and tactile inspection through the fracture site, this lesion was identified as a dense, spindle-shaped polymethyl methacrylate (bone cement) plug (Figures 2A,B). The plug completely occluded the medullary canal, and further reaming carries the risk of femoral blowout or iatrogenic fracture.

**Figure 3:**
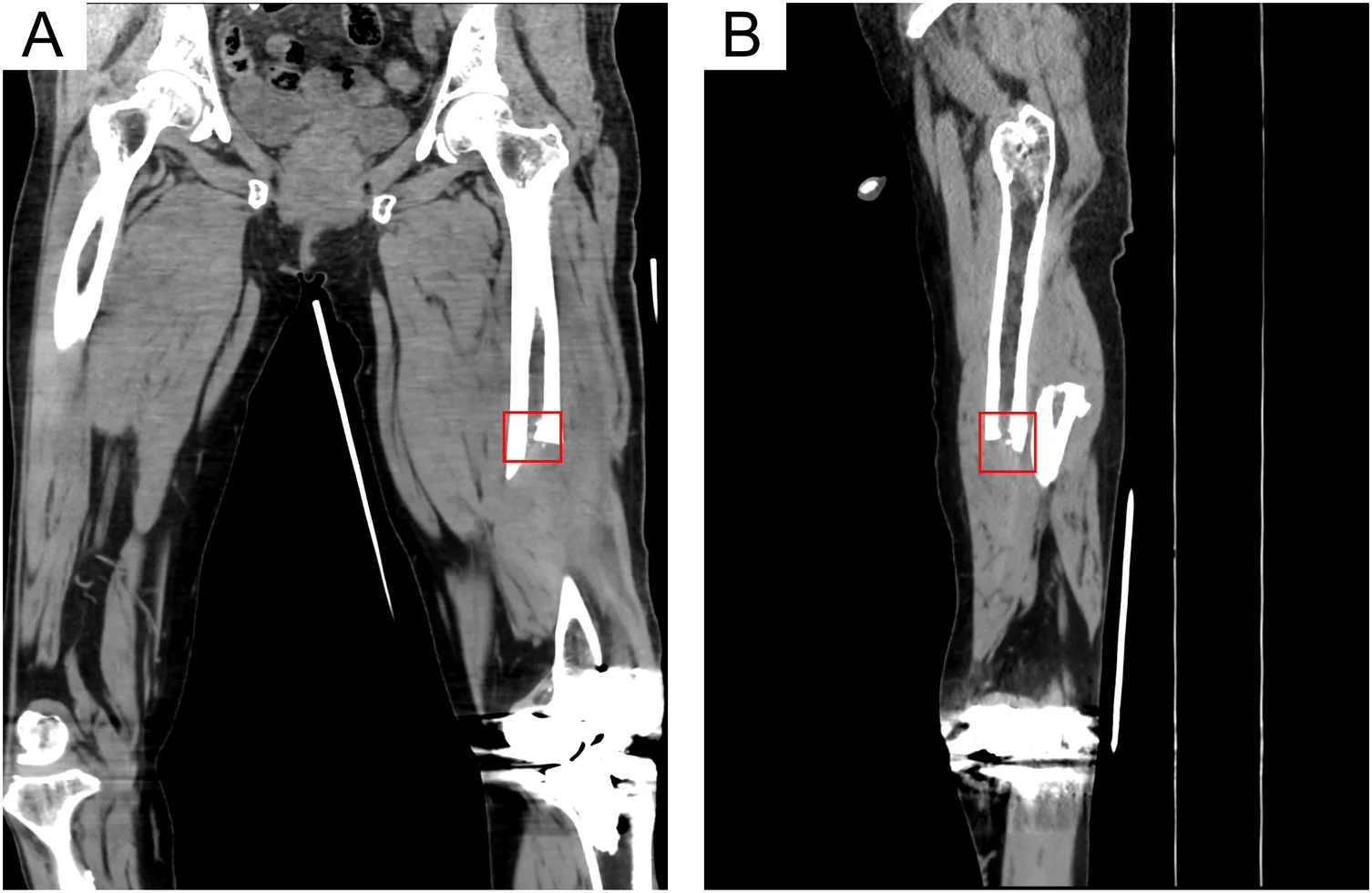
Preoperative CT imaging retrospectively identifying the bone cement plug. **(A,B)** Coronal and sagittal CT images, respectively, with red boxes highlighting the suspected cement plug within the medullary canal, which was not appreciated on initial interpretation but was later confirmed intraoperatively.

Faced with this unexpected crisis, the surgical team immediately suspended the original nailing procedure and initiated an emergency intraoperative contingency plan. The surgical approach was promptly transitioned from intramedullary fixation to extramedullary stabilization using a heavy-duty bone plate. Nevertheless, owing to the lack of preoperative preparation of an anatomical plate long enough to span the entire femoral segment, we had to resort to the use of an on-site shorter plate for connection. Despite that stable anatomical reduction was likewise accomplished, the outcome was less successful relative to the initial plan. The total surgical duration was approximately 130 min, with an estimated intraoperative blood loss of 500 mL. No iatrogenic fractures, cortical blowouts, or neurovascular injuries occurred during the operation. On the day of the procedure, the surgical team revisited the patient's preoperative imaging. Regrettably, no suspicious findings were noted on the plain radiographs; however, encouragingly, evidence of the cement encountered intraoperatively was observed on the CT scans (Figures 3A,B).

**Figure 4:**
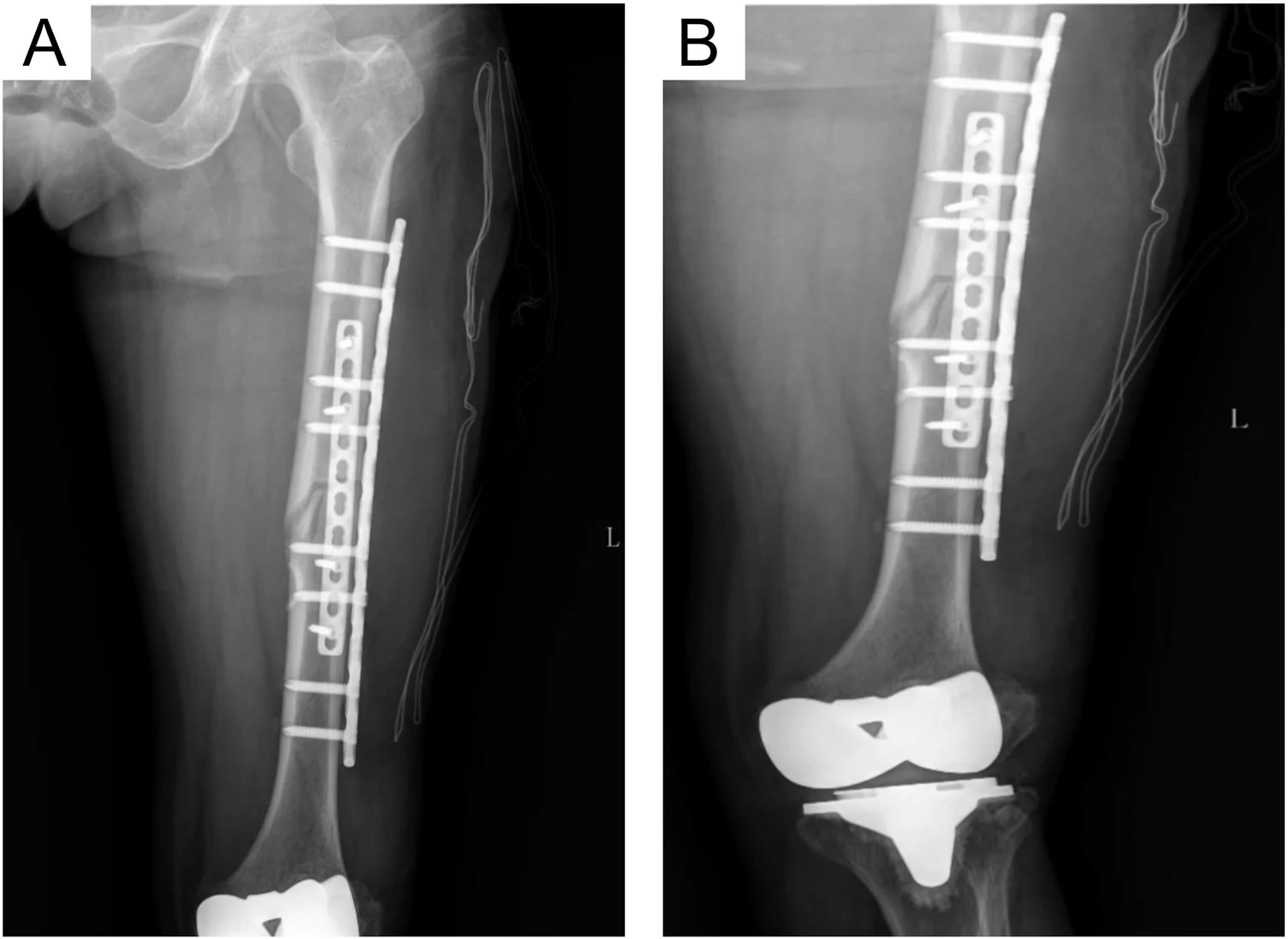
One-week postoperative full-length anteroposterior radiograph of the left femur. **(A,B)** The images demonstrate stable internal fixation and anatomical alignment achieved via extramedullary bone plating, successfully bypassing the fracture line and the occult bone cement plug site.

On the day of the procedure, a standardized postoperative care regimen was implemented, comprising intravenous antibiotic prophylaxis, low-molecular-weight heparin for thromboprophylaxis, and multimodal analgesia. On postoperative day 2, the patient began passive and active in-bed functional exercises under the protection of a functional knee brace. On postoperative day 5, under the guidance of a physical therapist, the patient started non-weight-bearing ambulation using a standard walker. One week postoperatively, follow-up radiographs showed very satisfactory alignment and apposition of the fracture ends, with the internal fixation plate in good position, successfully stabilizing the fracture fragments and spanning both the fracture line and the occult bone cement obstruction site (Figures 4A,B). Two weeks after surgery, the surgical incision achieved primary healing with no signs of superficial or deep infection, and the patient was discharged safely after suture removal. She then went abroad to a rehabilitation facility, strictly adhering to a progressive partial-weight-bearing to full-weight-bearing regimen. The patient was instructed to return for regular clinical and radiological follow-up.

## Discussion

The management of femoral shaft fractures in patients with a history of ipsilateral TKA is a technically demanding scenario that is becoming increasingly prevalent (7). While extramedullary plating and intramedullary nailing are both viable options, nailing is widely preferred for mid-shaft fractures because it avoids extensive periosteal stripping, minimizes blood loss, and offers superior biomechanical axial stability (8, 9). In addition, in the management of atypical femoral fractures, greater emphasis should be placed on preservation of lateral cortical blood supply and on avoidance of excessive intramedullary reaming. In the present case report, we must acknowledge that, upon retrospective review of the preoperative X-rays and CT images, subtle findings were identified that, in hindsight, may have suggested the presence of a cement plug. These findings had not been given due attention during the initial preoperative assessment, which ultimately led to the precarious intraoperative situation.

To the best of our knowledge, this is the first case report of a femoral fracture in a patient with a history of knee arthroplasty in which an occult intramedullary bone cement plug led to the failure of the planned intramedullary nailing. Polymethyl methacrylate (PMMA) bone cement is inherently radiolucent unless mixed with a radiopacifier such as barium sulfate or zirconium dioxide (10). While modern bone cements used in modern arthroplasties are routinely radiopaque, historical formulations, or cement restricted to distal medullary restriction plugs (often placed to prevent cement down-migration during pressurized component cementation), may contain lower concentrations of radiopacifiers or none at all. Furthermore, when a small or spindle-shaped cement mass becomes densely courted or closely opposed to the thick inner cortex of the femoral diaphysis, its attenuation profile on CT can closely match that of the surrounding host bone cortex, masking its presence on standard cross-sectional imaging (11). This poses a potential pitfall: although the medullary canal seems to be patent on multiplanar reformatted CT images, it is in fact mechanically occluded within the body. Furthermore, if time allows, comprehensive knowledge of the patient's prior knee replacement surgery history will allow clinicians to achieve more adequate preoperative preparation.

This case highlights the critical importance of intraoperative flexibility and crisis management in trauma surgery. Forcing an intramedullary reamer or nail against an unrecognized cement plug can result in severe iatrogenic complications, including femoral shaft splitting, thermal necrosis, or severe construct malposition. The immediate pivot to a bone plate allowed for a safe and stable construct, despite the logistical challenge of limited plate length within the emergency inventory. This emphasizes that for any patient presenting with a femoral fracture who has a history of knee or hip arthroplasty, surgeons must never assume canal patency based solely on reassuring preoperative images.

We acknowledge several limitations in this study. First, as a single-center case report, it provides a restricted level of clinical evidence and represents an isolated institutional experience. Second, the follow-up period presented is relatively short, focusing primarily on immediate postoperative recovery and short-term mobilization. Consequently, the long-term survival rate of the adjacent knee prosthesis, the ultimate quality and time to definitive fracture union, and any potential late complications (such as plate fatigue or nonunion due to the shorter plate length) require further long-term clinical and radiological observation. Furthermore, although the cement plug within the medullary canal was not identified preoperatively, subtle changes could still be discerned on the preoperative imaging findings in conjunction with the intraoperative observations. This alerts surgeons to the possibility of retained cement or other implant-related materials in patients with a history of knee arthroplasty, and underscores the importance of a more careful review of preoperative imaging studies.

In conclusion, patients with a history of knee arthroplasty presenting with an ipsilateral femoral shaft fracture can harbor occult, radiolucent, or masked medullary bone cement obstructions that escape detection on standard preoperative radiographs and CT evaluations. To mitigate the risk of severe intraoperative complications and avoid prolonged operative times, surgeons should routinely prepare multiple internal fixation modalities—including both intramedullary nails and extramedullary bone plates of varying lengths—prior to entering the operating room. Formulating reliable backup surgical strategies beforehand is essential to navigate these hidden anatomical pitfalls, minimize intraoperative risks, and ultimately optimize patient clinical outcomes.

## Data Availability

The original contributions presented in the study are included in the article/Supplementary Material, further inquiries can be directed to the corresponding author.
